# Insecticide exposure affects intergenerational patterns of DNA methylation in the Colorado potato beetle, *Leptinotarsa decemlineata*

**DOI:** 10.1111/eva.13153

**Published:** 2020-11-25

**Authors:** Kristian Brevik, Erika M. Bueno, Stephanie McKay, Sean D. Schoville, Yolanda H. Chen

**Affiliations:** ^1^ Department of Plant and Soil Science University of Vermont Burlington VT USA; ^2^ Department of Animal and Veterinary Sciences University of Vermont Burlington VT USA; ^3^ Department of Entomology University of Wisconsin Madison WI USA

**Keywords:** agriculture, DNA Methylation, epigenetics, evolution, insecticide, insecticide resistance, insects, transposable elements

## Abstract

Insecticide use is pervasive as a selective force in modern agroecosystems. Insect herbivores exposed to these insecticides have been able to rapidly evolve resistance to them, but how they are able to do so is poorly understood. One possible but largely unexplored explanation is that exposure to sublethal doses of insecticides may alter epigenetic patterns that are heritable. For instance, epigenetic mechanisms, such as DNA methylation that modifies gene expression without changing the underlying genetic code, may facilitate the emergence of resistant phenotypes in complex ways. We assessed the effects of sublethal insecticide exposure, with the neonicotinoid imidacloprid, on DNA methylation in the Colorado potato beetle, *Leptinotarsa decemlineata*, examining both global changes in DNA methylation and specific changes found within genes and transposable elements. We found that exposure to insecticide led to decreases in global DNA methylation for parent and F2 generations and that many of the sites of changes in methylation are found within genes associated with insecticide resistance, such as cytochrome P450s, or within transposable elements. Exposure to sublethal doses of insecticide caused heritable changes in DNA methylation in an agricultural insect herbivore. Therefore, epigenetics may play a role in insecticide resistance, highlighting a fundamental mechanism of evolution while informing how we might better coexist with insect species in agroecosystems.

## 1 INTRODUCTION

Insect herbivores in agroecosystems show a remarkable ability to rapidly adapt to novel forms of environmental stress, including synthetic insecticides (Brevik, et al., 2018). Current data suggest that at least six hundred arthropod species have developed resistance to over three hundred insecticidal active ingredients, with tens of thousands of reports of resistance worldwide, the vast majority since 1945 (Whalon et al., 2012). While it is often considered inevitable that insects will evolve resistance to insecticides (Alyokhin et al., 2015; Gould et al., 2018), how insect populations rapidly evolve this resistance remains poorly understood (Gould et al., 2018; Gressel, 2011; Oppold & Müller, 2017). Current evolutionary theory falls short of explaining the rapid evolution of insecticide resistance for multiple reasons (Laland et al., 2014). First, insect populations may not possess the standing variation to provide advantageous mutations to novel insecticidal toxins (Carrière & Tab ashnik, 2001). Second, new mutations may occur too infrequently to drive the pace of insecticide resistance (Karasov et al., 2010; Keightley et al., 2015), and the same insect species are repeatedly the first ones to develop resistance to new insecticides when they are introduced (Brevik, et al., 2018). If rates of insecticide resistance are based solely on our expectations of traditional Darwinian evolution, then repeated effects of extreme bottlenecks and low mutation rates should limit the ability for insects to develop resistance (Sax & Brown, 2000). On the other hand, insect herbivores have a long history of adapting to toxins found in plants and they may be preadapted to tolerate the insecticide classes that mimic plant compounds (Alyokhin & Chen, 2017). The paradox of insecticide resistance evolution is that despite experiencing strong selection that reduces insect population size and genetic variation, insects are still able to rapidly and repeatedly adapt.

Insecticide resistance occurs with the emergence of resistant phenotypes that can tolerate increasingly higher concentrations of insecticide. Much of our current understanding of insecticide resistance focuses on two major types of genetic mechanisms: *qualitative* changes, where mutations at a gene target site cause an insecticide to be less effective, and *quantitative* changes, such as increases in gene transcription that enhance the production of metabolic enzymes or increase the rate of toxin excretion due to accelerated metabolic pathways (Bass & Field, 2011; Ffrench‐Constant, 2013; Liu, 2015). While much of insecticide resistance literature has focused on qualitative changes because they are more straightforward to detect, quantitative changes in the expression of detoxification genes have been more important in conferring broad‐spectrum resistance (Cui et al., 2015; Li et al., 2007; Perry et al., 2011). Multiple studies have demonstrated how increased transcription of detoxification genes, such as P450s, glutathione S‐transferases, and esterases, underlies insecticide resistance (Ffrench‐Constant, 2014; Liu et al., 2015; Perry et al., 2011). In addition, researchers have observed that while insecticide resistance often increases in response to the frequency of insecticide use (Malekmohammadi, 2014; Tang et al., 2015; Yang et al., 2014), it is easily lost when insecticides are discontinued (Ffrench‐Constant et al., 1988; Foster et al., 2000). This phenotypic plasticity in response to changing environmental conditions may be due to epigenetic changes, which are able to change more rapidly than changes in the DNA sequence (Roberts & Gavery, 2012).

Epigenetics is the study of modifications that change how genes are expressed without changing the underlying DNA sequence of an organism. DNA methylation is a well‐documented mechanism of epigenetic inheritance that can influence phenotypic variation (Mendizabal et al., 2014). Methylation is the addition of a methyl group (CH3) to the 5‐carbon position of cytosines at CpG sites (Flores et al., 2013), which alters the level at which genes are transcribed. DNA methylation is widespread in insects (Glastad et al., 2011; Thomas et al., 2020), including beetles (Cunningham et al., 2015; Feliciello et al., 2015; Snell‐Rood et al., 2013). Methylation can occur throughout the genome, though its function may differ based on where it is located. In insects, the genomic regions that tend to exhibit DNA methylation are usually *within* genes and coding regions (Hunt et al., 2013a), while promoter regions remain largely clear of methylation. Increases in intragenic methylation in insect genes are associated with increased expression of those genes, as well as an increase in the number of alternative splice variants (Flores et al., 2012). When DNA methylation occurs in promoter regions, it is associated with gene silencing, as the methyl groups interfere with transcription machinery (Hunt et al., 2013b).

Emerging evidence suggests that insecticide exposure can directly and indirectly drive the evolution of insecticide resistance in agroecosystems via epigenetic processes (Brevik, et al., 2018). Pesticides may directly stimulate the expression of advantageous phenotypes, which may be underwritten by epigenetic modifications. Continued insecticide exposure in populations developing resistance would thus operate as "natural selection" and selectively increase the frequency of insect phenotypes that are adaptive to pesticides. Changes in the DNA methylation state of genes have been associated with insecticide resistance, and may be “a sensitive and reactive mode of action to enhance early‐on adaptation” (Oppold & Müller, 2017). For example, the green peach aphid, *Myzus persicae*, can gain insecticide resistance through the duplication of esterase genes and subsequent overexpression of esterases (Field et al., 1989).

However, Field et al. (1989) found that when methylation was lost on these genes, aphids became susceptible again, suggesting that methylation of esterase genes led to increased expression in aphids, and demethylation is associated with gene suppression. Importantly, methylation patterns were maintained over multiple generations, and the increased gene‐copy number was maintained, so it is possible that resistant aphids that had lost resistance through demethylation could quickly regain resistance through remethylation. In addition, insecticide exposure has been shown to alter patterns of global DNA methylation in bumblebees (Bebane et al., 2019) and honeybees (Paleolog et al., 2020), suggesting that insecticide exposure may interact with DNA methylation, which in turn shapes phenotypic responses to insecticide. Some changes in DNA methylation due to exposure to toxins or demethylating agents appear to be heritable in arthropods (Oppold et al., 2015; Vandegehuchte et al., 2010), but previous research has focused primarily on species such as *Daphnia magna*, which reproduce asexually, and it is unclear whether these changes persist through sexual reproduction. To date, no previous study has carefully examined how insecticide exposure influences heritable genome‐wide epigenetic modifications in insect herbivores.

If the epigenetic modifications that respond to environmental stress are heritable, they may play a role in rapid evolutionary change. For example, in the greater wax moth, *Galleria mellonella*, changes in DNA methylation and histone modifications facilitate the evolution of resistance to parasitic fungi by translating selection pressure into a heritable phenotype (Mukherjee et al., 2019). The parasitic wasp *Pimpla turionella* has been shown to modulate the epigenetics of host insects, decreasing DNA methylation, histone acetylation, and deacetylation, possibly leading to increased survival of larvae within hosts (Özbek et al., 2020). Beyond insects, it is thought that the evolution of finches in the Galapagos was mediated in part by changes in epigenetic marks, with genes associated with beak formation showing epigenetic changes (Skinner et al., 2014). Further inquiries into the role of epigenetics in the evolution of insecticide resistance may provide pathways to understanding the complex phenomenon of rapid evolution.

In addition to direct effects, the interplay between transposable elements and DNA methylation could influence insecticide resistance (Lippman et al., 2004; Xie et al., 2013). One of the primary roles of DNA methylation in eukaryotic genomes is to silence the activity of transposable elements (Zemach et al., 2010), which are mobile genetic elements that can either “jump” within the genome or "copy–paste" themselves, proliferating throughout the genome (Fablet & Vieira, 2011; Göke et al., 2016; Hosaka & Kakutani, 2018). TEs play essential roles in the structure and function of the genome, and the relationship is often symbiotic rather than parasitic (Dooner & Weil, 2013). TEs are responsible for many mutations within genomes and account for the bulk of the volume of most eukaryotic genomes (Fedoroff, 2012). They are also likely responsible for some of the most important structural elements in the genome, such as introns (Huff et al., 2016). TEs generate genetic variation (Kidwell & Lisch, 1997) via a number of mechanisms, including inserting upstream of a gene and altering gene expression levels (Daborn, 2002) and duplicating genes (Berger et al., 2016), both of which have been implicated in the evolution of insecticide resistance. Changes in the DNA methylation state of TEs can also be associated with rapid evolution, and there is considerable evidence that stress, such as exposure to toxins, can lead to the mobilization of transposable elements (Cappucci et al., 2019; Chadha & Sharma, 2014; Horváth et al., 2017). In insects such as *Drosophila melanogaster*, exposure to heat stress is associated with increased rates of transposable element activation, which appears to be due to interactions between heat shock proteins, RNA, and transposable element suppression (Specchia et al., 2010).

The Colorado potato beetle, *Leptinotarsa decemlineata* (Coleoptera: Chrysomelidae), is an important model for the study of rapid adaptation in insects. The beetle appears to evolve resistance at a greater rate compared with other insects (Brevik, et al., 2018). It has evolved resistance to every insecticide used against it, currently over 55 insecticides (Alyokhin et al., 2008). With a global distribution that encompasses the entire potato‐growing area of the Northern Hemisphere (Weber, 2003), the beetle has adapted to a remarkable range of climates (Lehmann et al., 2014), host plants (Jacques, 1988, Crossley, Chen, Groves, & Schoville, 2017), and insecticides (Alyokhin et al., 2008; Argentine et al., 1989; Zhu et al., 1996). Before encountering the potatoes planted by European settlers in what is now the United States, the beetle fed on several plant species in the genus *Solanum*, including buffalo bur, *Solanum rostratum* (Jacques, 1988). The beetle was first reported to have expanded its host range to feed on the potato, *Solanum tuberosum*, in 1859 in Nebraska (Walsh 1865). Following its invasion into Europe and continuing into Asia, the beetle has evolved rapidly to face a number of novel stressors and environments, including dozens of insecticides and colder northern climates (Alyokhin et al., 2015; Grapputo et al., 2005). The beetle evolves resistance to new insecticides in an average of 34 generations, or about 10 years (Brevik, et al., 2018). Therefore, the beetle's widespread distribution, adaptability, and impact on potato make this species ideal for understanding how the effects of insecticide exposure shape the responses of insect herbivores to the management of agroecosystems.

To determine whether exposure to insecticide leads to changes in DNA methylation in the Colorado potato beetle, we used an experimental approach to test whether insecticide exposure altered heritable patterns of DNA methylation in the Colorado potato beetle across multiple generations. By sequencing the DNA epigenome of exposed and F2 beetles, we tested whether the epigenetic responses could be heritable. First, we tested how exposure to a common neonicotinoid insecticide, imidacloprid, influenced patterns in global DNA methylation in the parent and F2 generations. Our study allowed us to test for direct effects of imidacloprid on DNA methylation levels on the exposed generation, and whether these patterns persisted through two generations. Second, we tested where differential methylation occurred in the genome, by looking at each site (CpG nucleotide) that was found to be differentially methylated in beetles exposed to insecticide treatments. This analysis examined which differentially methylated sites were associated with (a) annotated genes, (b) the flanking regions of annotated genes, or (c) annotated transposable elements. Together, these analyses provide insight as to how DNA methylation may play a role in the rapid adaptation of the Colorado potato beetle to insecticides.

## 2 MATERIALS AND METHODS

### 2.1 Insect rearing

We started a beetle colony by collecting 50 adult beetles from three organic potato farms in Vermont in June 2015 and pooling them into a single colony. We chose to use imidacloprid, a neonicotinoid insecticide, because it is the most widely used insecticide currently deployed against the beetle (Mota‐Sanchez et al., 2006). In order to minimize the possibility that that the collected beetle populations had been previously exposed to imidacloprid, we carefully selected farms that have been certified organic since the early 1990s, before the introduction of imidacloprid. We reasoned that prior exposure of a beetle population to imidacloprid may have been selected for higher overall resistance, which may influence epigenetic responses in this study. However, organic growers can use spinosad under organic agriculture certification standards, which shows a low‐to‐moderate cross‐resistance with imidacloprid (Mota‐Sanchez et al., 2006). Therefore, the field‐collected beetles likely have a low‐to‐moderate level of prior resistance to imidacloprid. In order to minimize any maternal effects arising from previous environmental exposure, the colony was reared for four generations before the experiment took place. We maintained the beetle colonies on live potato plants at 24°C (16:8 LD) in 60 cm × 60 cm × 40 cm cages using potato plants. The potato plants (*Solanum tuberosum* L., var. Kennebec) were in Fafard 3B potting mix (Fafard, Agawam, MA, USA) in 10.2‐cm square pots in the greenhouse for 6–8 weeks. Plants were fertilized with a liquid fertilizer twice a week during watering (17‐4‐17, N‐P‐K). Plants were grown for 6–8 weeks before they were fed to the beetles. Eggs were removed from each colony twice a day and moved to smaller rearing cages to minimize cannibalism and prevent overlap of generations.

### 2.2 Study design

To determine whether insecticide exposure changed DNA methylation patterns in the Colorado potato beetle, we exposed beetles to sublethal dosages of the neonicotinoid insecticide imidacloprid. We sampled adult beetles from each treatment during the exposed and F2 generations, and sequenced the beetles using a whole‐genome bisulfite sequencing (WGBS) approach to assess changes in DNA methylation throughout the genome. Given that environmental conditions are thought to influence patterns of DNA methylation independent of ancestry, we elected to use a mass‐rearing approach of selecting individuals from a colony, rather than following a pedigree breeding approach to test for the possibility of intergenerational (F2) inheritance. By selecting random individuals from the colony, we used a more conservative approach by incorporating greater level of heterogeneity, allowing us to detect whether the patterns of DNA methylation were similar across beetle individuals from the same treatment, regardless of ancestry.

### 2.3 Treatments

We selected insecticide treatments that would impose different levels of stress. The four treatments varied in their dosage and toxicity (1 ppm imidacloprid, 0.1 ppm imidacloprid, 1 ppm imidacloprid analog, and water control). Technical grade imidacloprid was obtained from Bayer. We first calculated the LC_10_ dosage by determining the dosage that caused 10% mortality. Using previous data on baseline imidacloprid susceptibility of 134 geographically discrete populations (Olson et al., 2000), we expected that a 1 ppm exposure would be a sublethal dose because it was a third of the dose of the lowest reported LC_50_. We verified the susceptibility of the local population by pipetting 1 ppm dose onto 20 beetles, each was exposed to this dose, across 10 replicates. On average, we found that an average of two beetles per exposure group died. Our goal was to develop treatments that presented different levels of exposure to the active compound rather than accurately gauge the true LC concentration values for the population. Therefore, we opted for multiple replicate assays using small populations rather than the typical large population assays of 100 individuals per population. We estimated that the LC_10_ was at 1 ppm dosage, which would deliver a stressful, yet sublethal, dose. We reasoned that the 0.1 ppm dose, which would deliver 10% of the active compound, the LC_10_, would be even less stressful. Even though all concentrations below the LC_50_ level are considered to be sublethal, the 1 ppm treatment was intended to be fully sublethal to all beetles (Olson et al., 2000). We applied a 1 µl droplet of insecticide treatment onto dorsal side of the thorax of 50 randomly selected fourth‐instar larvae per treatment.

**Figure 1:**
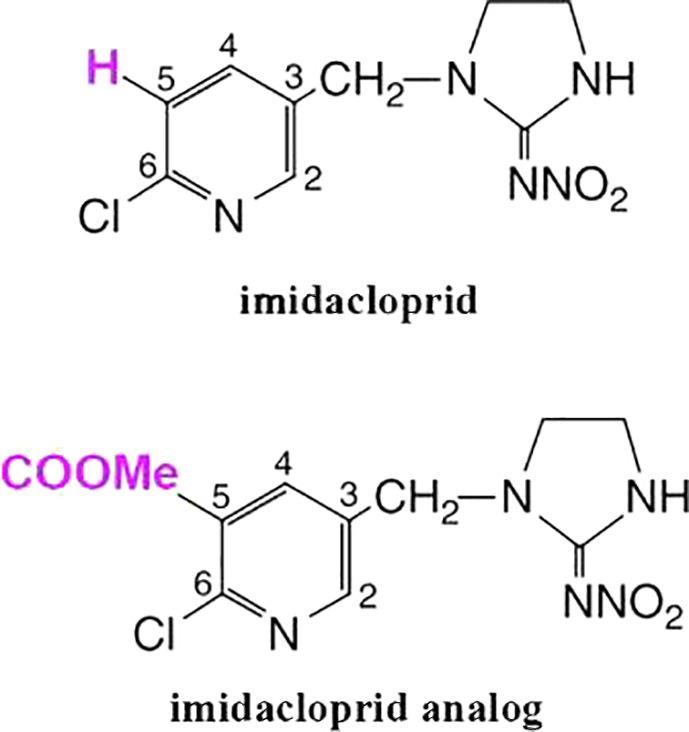
Comparison of the chemical structure of imidacloprid with the chemical structure of the imidacloprid analog used in this study, where a hydrogen has been replaced with a methoxycarbonyl group. Differences highlighted in pink

In order to control for chemistry of the compound itself, we used an analog for the insecticide imidacloprid, the chemical *1‐(6‐chloro‐5‐methoxycarbonylpyridin‐3‐ylmethyl)‐2‐nitroiminoimidazolidine*. Analogous to cage‐controls in ecological experiments, the imidacloprid analog was a compound found to be considerably less toxic than imidacloprid, which could allow us to separate the effects of the compound on DNA methylation from the degree of toxicity. The imidacloprid analog was custom‐synthesized to be chemically similar to imidacloprid, but the slight difference in molecular structure minimized the toxicity (Kagabu et al., 2007). Figure 1 shows how the molecular structure of imidacloprid and the imidacloprid analog differ, where imidacloprid has a hydrogen, and the analog contains a methoxycarbonyl group (‐COOMe). Although we did not verify that the methoxycarbonyl group directly reduced toxicity, we found that none of the larvae exposed to this treatment died. We used the imidacloprid analog to separate the effects of the *toxicity* of imidacloprid from the effects of mere *exposure to a chemical of this nature*. After the exposure to each treatment, the surviving larvae from each treatment were used to found separate colonies that propagated over four additional generations. For genome sequencing, we sampled the adults from each treatment for four subsequent generations, including the exposed generation. Due to budget limitations, only the exposed and F2 generations were included in this study.

### 2.4 DNA extraction and sequencing

In both the exposed and F2 generation, bisulfite sequencing was conducted on the DNA extracted from 16 exposed beetles, 4 from each treatment, for a total of 32 individuals. We extracted DNA from half of the thorax and abdomen of adult beetles for genomic DNA sequencing using the Omega Bio‐Tek E. Z. N. A. Mollusc DNA Kit (Omega Bio‐Tek, Norcross, GA). Following the genomic DNA extraction, we verified DNA quality and concentration using agarose gel electrophoresis and Qubit prior to library construction. After quality testing, positive control DNA was added and the DNA was fragmented into 200‐400bp using Covaris S220.

Sequencing adapters were ligated to the DNA fragments. DNA libraries were bisulfite‐treated using EZ DNA Methylation Gold Kit (Zymo Research). Library concentration was quantified by Qubit 2.0, and then was diluted to 1 ng/µl before the insert size was checked on Agilent 2100 and quantified using qPCR. Libraries were then pooled, and then, paired‐end sequencing was conducted via Illumina, with 150 base pair reads.

### 2.5 Analysis

For analysis, we relied on the packages Bismark (Krueger & Andrews, 2011) and methylKit (Akalin et al., 2012) to examine which cytosine nucleotides exhibited differential methylation between treatments. Sequenced reads were checked for quality using FastQC (Andrews, 2010), adapters were trimmed, and deduplication was done using Bismark and samtools (function *merge*). Sequenced reads were mapped to the *L*.* decemlineata* reference genome (v. 1.0) using Bismark (*default parameters*). Each site had a mean coverage of 60.75.

To assess differential methylation between treatments, we used the R package methylKit version 3.11, which provided assessments of which sites exhibited differential methylation (function *processBismarkAln*, parameters: *read*.*context*="*CpG*”, *nolap* =*FALSE*,*mincov* =*10*,*minqual* =*20* and function *filterByCoverage*, parameters: *lo*.*count* =*10*,*lo*.*perc* =*NULL*,*hi*.*count* =*NULL*,*hi*.*perc* =*99*.*9*). To determine whether changes in methylation sites were consistent between generations, we utilized an ANOVA approach (functions *lm/ANOVA*, from package *stats v3*.*6*.*2*) comparing differential methylation within and between treatments. Global DNA methylation was calculated as methylated cytosines as a percentage of all cytosines. For all tests, a minimum change of 10% methylation level with a Q‐value cutoff of 0.01 was used (function *getMethylDiff*, parameters: *difference *=* 10*,*qvalue *=* 0*.*01*). In order to assess the effect of treatment, generation, and treatment x generation on CpG methylation, we conducted ANOVA tests in R (package *stats v3*.*6*.*2*, function *aov*).

In order to find which differentially methylated sites were associated with certain genomic features (gene annotations, 2 kb gene flanking regions, and transposable elements), we used the package bedtools (*v*.*2*.*29*.*2*) functions "flank" and "intersect." Gene annotations were used from the Colorado potato beetle (version 1.0) reference genome (Schoville et al., 2018), and transposable elements were annotated using the discovery pipeline described in (Brevik *et al*.*in prep*) using RepeatModeler (version 1.0.8) (Smit, Hubley & Green, 2013-2015) (using parameters ‐*dir* Custom *‐pa 20*), We then used RepeatMasker (*parameters*:*‐s ‐pa 18 ‐gff*) to detect the locations of these 334 "active" transposable elements in the genome. To determine whether differentially methylated regions occur more frequently within transposable elements compared with genome‐wide methylation, we used a chi‐square test in R (function *chsq*.*test*, parameters *default*).

## 3 RESULTS

**Figure 2:**
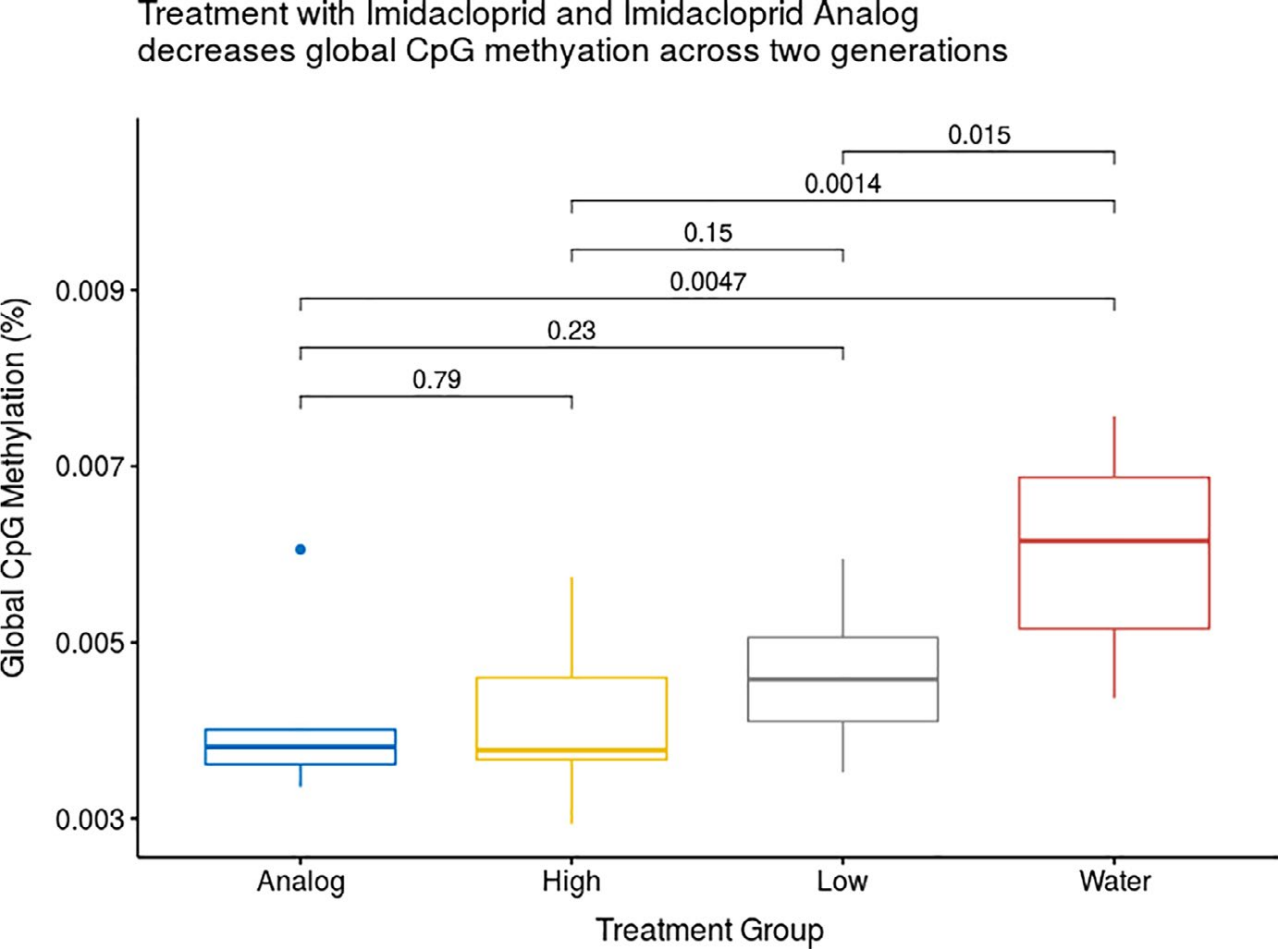
ANOVA results of comparison of global DNA methylation percentage of treatment groups with water control. The three treatments (analog, high imidacloprid, and low imidacloprid) differ from the water control, but not from each other

**Figure 3:**
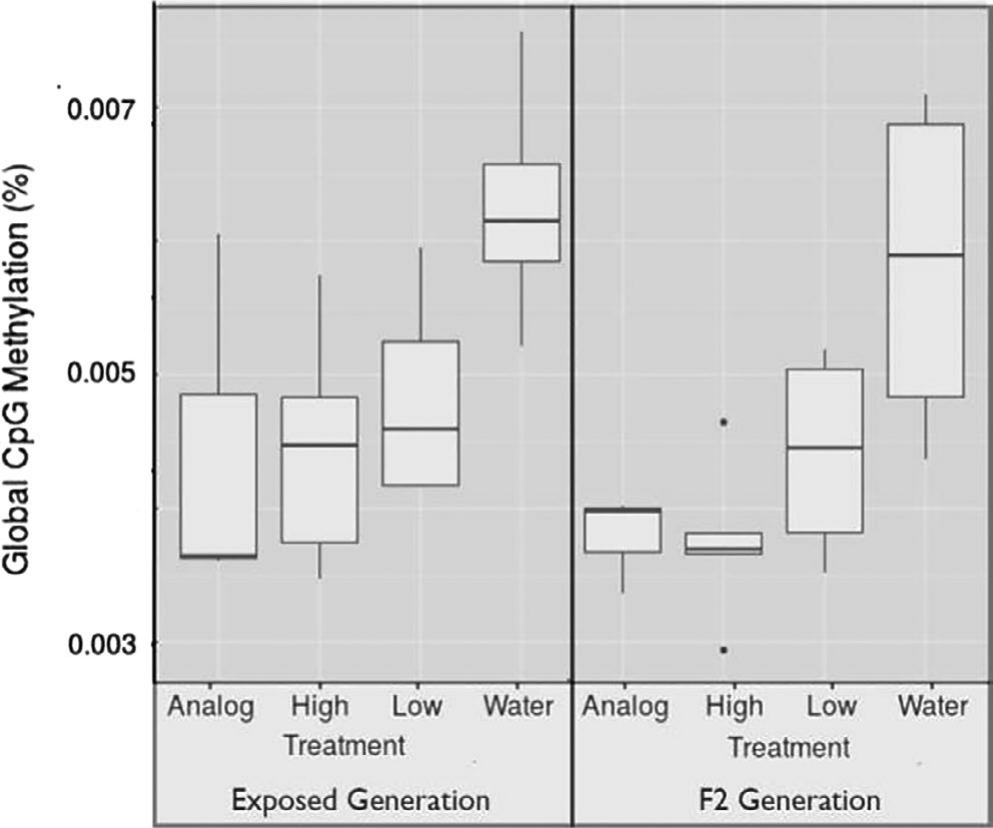
Comparison of global DNA methylation percentage of treatment groups with water control, separated by generation. The three treatments (analog, high imidacloprid, and low imidacloprid) differ from the water control, but not from each other, and the effects do not differ between generations

All beetles exposed to insecticides (1 ppm imidacloprid, 0.1 ppm imidacloprid, and 1 ppm analog) showed a decrease in global DNA methylation compared with the beetles exposed to water. The decrease in global DNA methylation was maintained across two generations until the F2 generation (Figure 2, p‐values in figure). Overall, global DNA methylation was quite low. On average, 0.047% of cytosine nucleotides were methylated per beetle, with a range of 0.029%–0.075%. However, exposure to insecticides decreased methylation from 0.06% (0.043%–0.075%) in the control to an average of 0.042% (0.029%–0.06%) in the treated groups, a difference of approximately 0.02%. There was no effect of beetle generation on global DNA methylation, and beetle generation and treatment did not significantly interact. Figure 3 shows differential methylation in the two generations separately. Because the ANOVA test showed that the three treatments were each significantly different from the water control but not from each other, we compared all three insecticide treatments together with the control in subsequent analyses. Analysis of differential methylation within each treatment verified that variation in differential methylation was smaller within each treatment than between treatments (*F* = 282.08, *p* < .001), and has consistent effects across generations.

**Figure 4:**
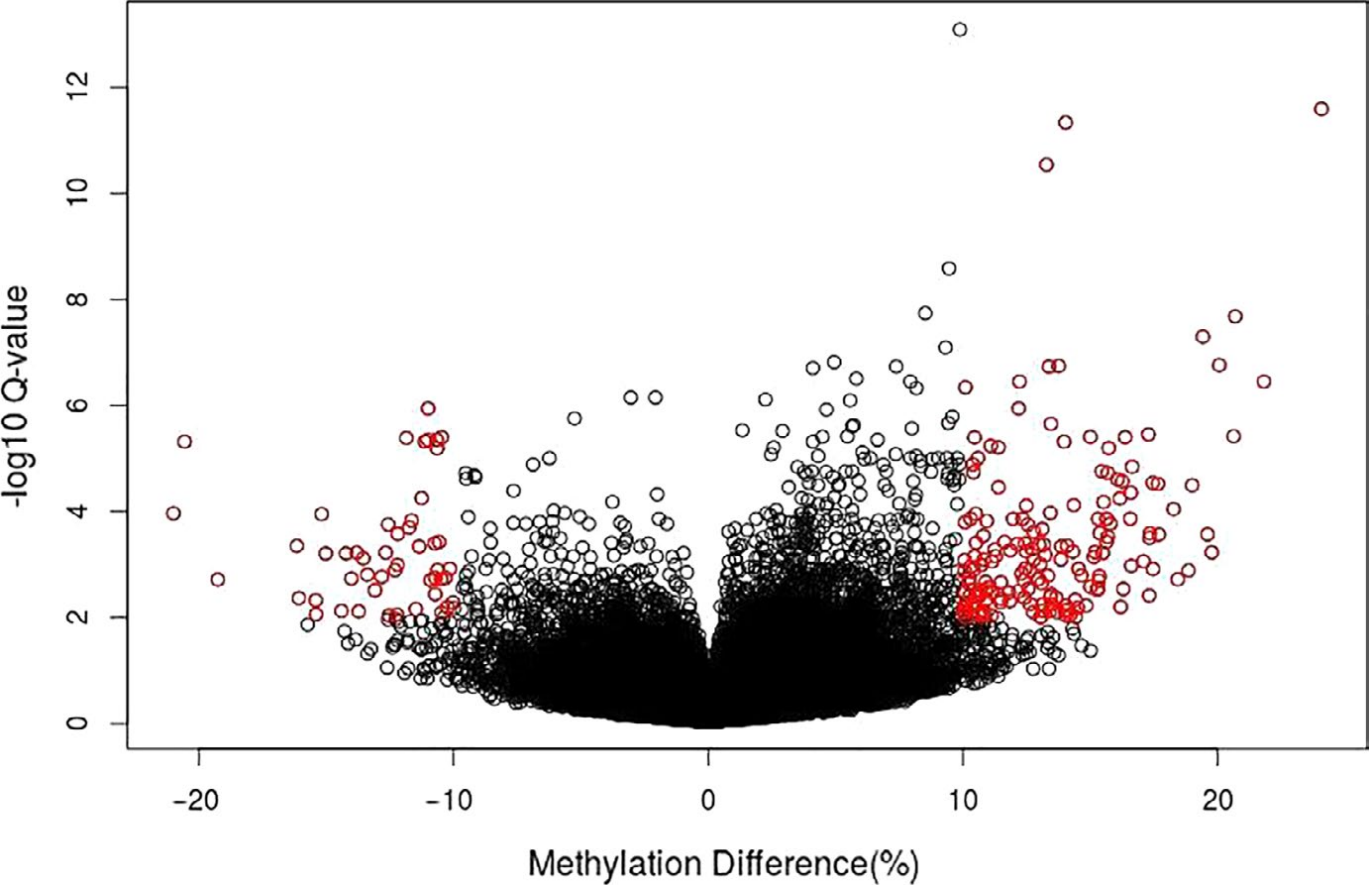
Volcano plot showing methylation difference compared with Q‐value. Red dots are those selected for further analysis, using a minimum change of 10% methylation level with a Q‐value cutoff of 0.01

In comparing the three insecticide treatments with the water control, we found that 221 sites showed differential methylation of 10% or more, using a Q‐value cutoff of 0.01 (Figure 4). These values were further examined as highly significant sites with both notable changes in methylation and confidence in our findings. Of the 221 differentially methylated sites common to all beetles exposed to insecticide, nine of these sites were found within four gene annotations in the genome (Table 1), with multiple sites per annotation. Two of these genes are cytochrome P450s that are already associated with resistance, *LDEC011287* and *LDEC015052*. *LDEC011287* contained three differentially methylated sites, and *LDEC015052* had two. All five sites showed increased levels of methylation. One of the remaining two genes is uncharacterized in the current genome annotation and showed one site of increased methylation and one site of decreased methylation. The fourth gene is a putative cyclin‐dependent kinase, and both methylation sites found within this gene showed decreased methylation. Among the remaining differentially methylated sites, three were found within the 2‐kb flanking regions of annotated genes (Table 1). Two of these sites occurred within the flanking region of the same putative cyclin‐dependent kinase, *LDEC015089*, and the third occurred within the flanking region of a glycoside hydrolase. Close to 39% (86) of the differentially methylated sites fell within 47 transposable element annotations, with some transposable elements containing multiple variable methylation sites (Table 1). A chi‐square test shows that differentially methylated sites were overrepresented in transposable elements compared with the genome as a whole (chi‐square = 5.6365, *p*‐value < .05). Most of these transposable elements were LINE elements, though a number of other types are also represented.

**Table 1:** Annotated genes, gene flanking regions, and transposable elements that were found to contain differentially methylated sites when all three treatments were together compared with control

Gene name	Gene function	No. of differentially methylated sites	Direction of change
a) Genes within which differentially methylated sites were found			
*LDEC011287*	Cytochrome P450 (Tribolium castaneum homologue)	3	Increased
*LDEC015052*	Cytochrome P450 (Tribolium castaneum homologue)	2	Increased
*LDEC004892*	Uncharacterized	2	One decreased, one increased
*LDEC015089*	Putative cyclin‐dependent kinase	2	Decreased
b) Genes where differentially methylated sites were found within 2‐kb flanking regions			
LDEC015089	Putative cyclin‐dependent kinase	2	One decreased, one increased
LDEC004246	Glycoside hydrolase 45	1	Decreased
Transposable element type	No. of differentially methylated sites	Direction of change	

c) Transposable elements where differentially methylated sites were found			
LINE/LOA	1	Increased	
LINE/L2	1	Decreased	
LINE/Penelope	1	Decreased	
LINE/Tad1	1	Decreased	
DNA/PiggyBac	1	Decreased	
LTR/Gypsy	1	Decreased	
LINE/L2	5	Decreased	
DNA/hAT‐Charlie	1	Decreased	
LINE/L2	2	Decreased	
LINE/Jockey	1	Increased	
LINE/Jockey	1	Increased	
LINE/Penelope	1	Decreased	
DNA/TcMar‐Tc1	1	Decreased	
DNA/PiggyBac	1	Decreased	
LINE/L2	3	Decreased	
LINE/CR1	4	Decreased	
LINE/L2	1	Increased	
LINE/L2	1	Decreased	
LINE/L2	3	Decreased	
LINE/L2	3	Decreased	
LINE/L2	1	Decreased	
LINE/L2	1	Decreased	
LINE/L2	1	Decreased	
LINE/CR1	2	Decreased	
LINE/L2	1	Increased	
LINE/CR1	1	Increased	
LINE/L2	2	Decreased	
LTR/Gypsy	1	Increased	
LINE/L2	1	Decreased	
LINE/L2	3	Decreased	
LINE/L2	1	Decreased	
LINE/L2	1	Increased	
LINE/L2	1	Decreased	
DNA/TcMar‐Tc1	1	Decreased	
LTR/Gypsy	2	Decreased	
LINE/Jockey	1	Decreased	
LINE/L2	1	Decreased	
DNA/hAT‐Charlie	1	Decreased	
LINE/Jockey	3	Increased	
LINE/L2	1	Decreased	
LINE/L2	1	Decreased	
DNA/TcMar‐Tc1	2	Decreased	
LINE/L2	1	Decreased	
LTR/Copia	4	Decreased	
LTR/Copia	5	Decreased	
LTR/Copia	4	Decreased	
LINE/L2	3	Decreased	

**Figure 5:**
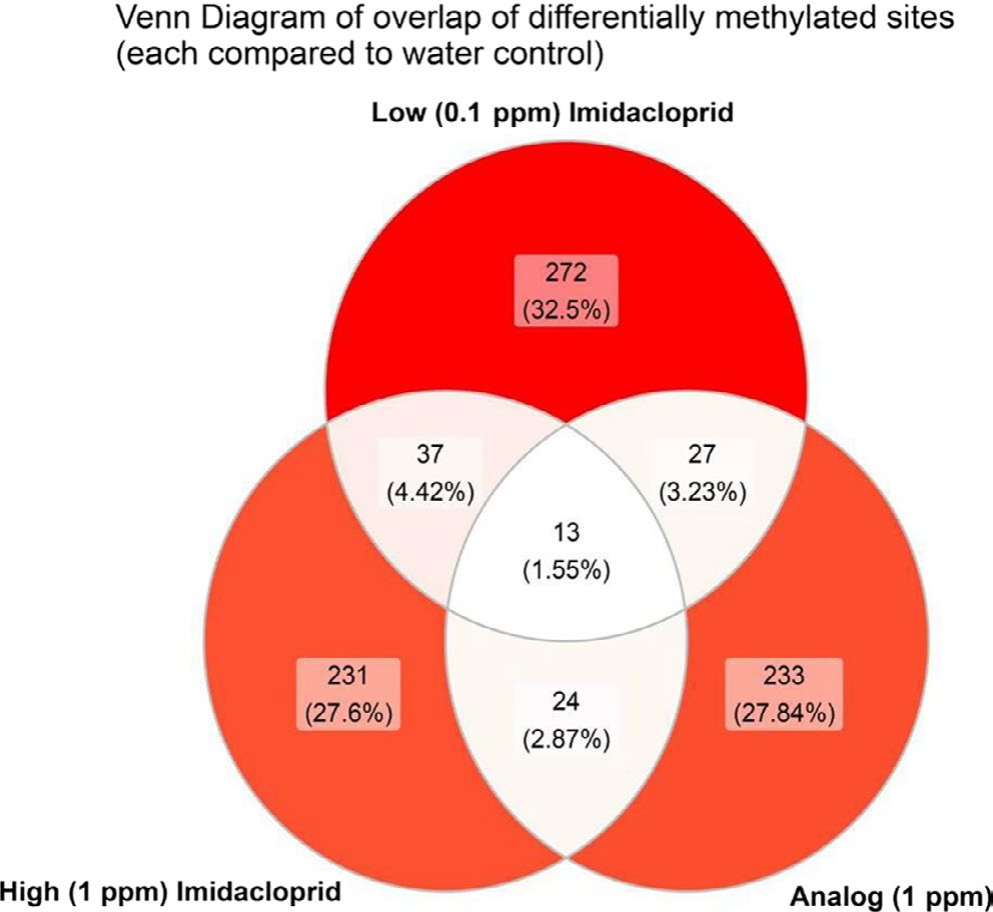
Venn diagram illustrating the overlap of differentially methylated sites between treatments (imidacloprid analog, high imidacloprid, and low imidacloprid), each compared with a water control

Although beetles exposed to imidacloprid showed a decrease in global methylation, the location of the individual methylation sites varied by treatment. When each analysis was independently compared with the water control, we found that only 1.55% or 13 sites showed a similar pattern in differential methylation across all three treatments (Figure 5). While none of these 13 sites overlapped with any gene annotations in the genome or with any flanking regions for gene annotations, three of them were found within LINE transposable element annotations (Table 2).

**Table 2:** Annotated genes, gene flanking regions, and transposable elements that were found to contain differentially methylated sites when each treatment was assessed independently and the results were reconciled

Transposable element type	No. of differentially methylated sites	Direction of change
Transposable elements where differentially methylated sites were found		
LINE/CR1	1	Decreased
LINE/L2	1	Decreased
LINE/L2	1	Decreased

## 4 DISCUSSION

The emerging perspective in environmental epigenetics is that environmental exposure to a range of chemicals can cause lasting heritable effects. Environmentally induced changes in epigenetics can lead to a number of phenotypic effects that persist through generations, from disease etiology (Nilsson et al., 2018), to adaptive responses to environmental change (Thiebaut et al., 2019). These epigenetic changes can influence developmental bias, phenotypic plasticity, and niche construction, contributing to evolutionary dynamics (Jeremias et al., 2018). Indeed, it is thought that environmentally induced changes in epigenetics may have contributed to the evolution and diversification of Darwin's finches (Skinner et al., 2014). DNA methylation has been shown to be heritable across multiple generations, which may lead to sustained adaptation.

Also, DNA methylation influences critical patterns of gene expression in insects as well (Glastad et al., 2014). In social insects, gene expression modulated by DNA methylation plays a role in the determining of caste (Glastad et al., 2011; Weiner et al., 2013), while in other species, changes in DNA methylation are associated with changes in sensitivity to toxic chemicals (Field et al., 1989; Oppold et al., 2015). Insight into these mechanisms provides novel ways of understanding the rapid emergence of insecticide resistance in insects and may help to resolve the paradox of insecticide resistance.

We show that insecticide exposure can influence the patterning of heritable epigenetic modifications in the Colorado potato beetle. Exposure to insecticides decreased global methylation in the beetle, highlighting a possible apparent trade‐off between detoxification and epigenetic regulation. Toxin exposure may reduce global DNA methylation (Hunter et al., 2014; Oppold et al., 2015), and one possible mechanism is due to competition between biochemical pathways. DNA methylation of genomic DNA is dependent upon the availability of methyl groups and S‐adenosylmethionine (Lee et al., 2009). S‐ adenosylmethionine provides the methyl groups for methyltransferases to methylate DNA. Glutathione, which is an antioxidant that conjugates with xenobiotic toxins, requires homocysteine, which is also needed as a precursor for S‐adenosylmethionine (Enayati et al., 2005). In the presence of toxins, detoxification becomes imperative and depletes homocysteine (Oppold & Müller, 2017), which may lead to a lack of S‐adenosylmethionine available for DNA methylation and a corresponding decrease in DNA methylation in the genome (Lee et al., 2009; Oppold & Müller, 2017). In this case, it could be that the biochemical pathways that are involved in detoxification are depleting the biochemical precursors that are needed to methylate DNA. Given the minimal overlap across treatments in the differentially methylated cytosines, our data suggest that changes in DNA methylation may occur randomly within the genome.

The effects of insecticide exposure were consistent between the two generations of tested individuals, suggesting that patterns of DNA methylation could be heritable as seen in Figures 2 and 3. While this phenomenon has been shown in asexually reproducing species (Vandegehuchte et al., 2010), this consistent pattern shown in our study suggests that environmentally induced phenotypes may persist through generations in a sexually reproducing insect. These patterns may play a role in rapid evolution of these species, as intergenerational or transgenerational inheritance of epigenetic effects may facilitate the emergence of resistant phenotypes, and even short‐term phenotypic changes may have significant implications for agriculture. This is particularly relevant for insects that are exposed to insecticides, such as those found in agroecosystems—and those with whom humans often find themselves in conflict with. Interestingly, we did not find a clear relationship between insecticide toxicity and global DNA methylation. Despite the reported lack of insecticidal activity for the imidacloprid analog (Kagabu et al., 2007), it caused similar changes in global DNA methylation as the more toxic imidacloprid. Furthermore, even the 0.1 ppm dosage caused a similar effect. The parallel responses across all insecticide treatments suggest that acute toxicity may not be as important as mere exposure to novel compounds. Additionally, all treatment doses led to a similar decrease in global DNA methylation, suggesting that very low doses (much lower than many insects receive in the field) may play a role in causing changes in methylation (Desneux et al., 2007). Therefore as suggested by Lee et al. (2009), simply the exposure to novel chemicals may cause long‐lasting and unpredictable effects within the genomes of exposed individuals.

The specific genes where DNA methylation changed provide support for a role of methylation in insecticide resistance. Exposure to imidacloprid increased methylation of cytochrome P450s, which is one of the main groups of enzymes associated with detoxifying insecticides in insects (Feyereisen, 1999; Liu et al., 2015; Puinean et al., 2010; Scott, 1999). Some examples of insecticide resistance in the Colorado potato beetle are due to either mutations in cytochrome P450 genes or changes in the levels of transcription of these genes (Clements et al., 2016). Glycoside hydrolases are genes involved in the breakdown of glycoside, which are compounds found in plants, and are found only in *Phytophaga* (leaf‐eating beetles) among insects (Busch et al., 2019), a clade of plant‐eating beetles, which includes the *L*.* decemlineata*. This may be significant because many of the genes that have evolved to deal with plant toxins are able to be used by the beetle to adapt to the toxins found in insecticides (Zhu et al., 2016). The regulation of cyclin‐dependent kinases may be more challenging to understand, because these genes are involved in regulating the cell cycle (Malumbres, 2014), though it is notable that this is the one type of gene that showed changes in methylation in both the gene and neighboring flanking regions. Together, the narrow subset of genes that showed changes in DNA methylation levels is surprising, and further inquiry on these and similar genes may yield insight into how these genes are expressed and how changes in DNA methylation influence beetle phenotypes.

It is remarkable that among the 221 sites that showed changes in DNA methylation, many fell within transposable elements. Given that approximately 17% of the genome is made of up TEs (Schoville et al., 2018) but 39% of differentially methylated sites from this study are found within TEs, it appears that TEs may be subject to a disproportionate amount of differential methylation. While the overrepresentation of TEs as sites for differential DNA methylation could be influenced by the assembly of the reference genome, it may also be possible DNA methylation within transposable elements can be associated with exposure to insecticides.

Transposable elements are commonly suppressed by DNA methylation to prevent them from causing mutations (Lippman et al., 2004). If insecticide exposure alters the DNA methylation of transposable elements, they may be more able to generate mutation in an affected insect, and these mutations may be associated with resistance. Indeed, in *D*.* melanogaster*, repeated insertions of transposable elements within stress‐response genes may be associated with increased stress tolerance (Merenciano et al., 2016). Our results lend support to a pathway by which changes in genome regulation may drive a dynamic interplay between epigenetics and transposable elements, which may contribute to the development of insecticide resistance.

Our study was limited for several reasons. We did not track the pedigree of each exposed beetle, but instead looked at colony‐wide effects, which limited our ability to assess the maintenance of DNA methylation changes at specific sites. We also did not link changes in methylation to either gene expression or phenotypic changes, which would have provided a more robust assessment of how changes in DNA methylation due to insecticide exposure impact the fitness of beetles when encountering insecticides or other stressors. Nevertheless, we provide initial confirmation of the presence of DNA methylation in the Colorado potato beetle and how insecticide exposure causes changes in methylation in genes associated with resistance. In addition, we show that these changes in DNA methylation can last for at least two generations, indicating how epigenetic variation can be heritable within a population.

We suggest that complex interactions between insecticide exposure, transposable element activity and epigenetics may play a role in insecticide resistance. These elements together may contribute to the ability of insects to rapidly evolve in agroecosystems by explaining how our expectations surrounding bottlenecks, low mutation rates, and strong selection do not always line up with the rate of evolution of insecticide resistance. Further research incorporating more analyses is necessary to validate these results—including transcriptome sequencing and phenotypic assays to determine whether changes in DNA methylation are associated with changes in transcription and insecticide resistance. Future research may also choose to focus on specific genes, such as cytochrome P450s, to more fully assess and understand the nuances of how changes in DNA methylation influence the genes associated with insecticide resistance and other stressors. Our results provide a strong imperative for comprehensive, multigenerational longitudinal studies that follow populations of insects after insecticide exposure, monitoring epigenetic changes, gene expression changes (including transposable element expression), and whole‐genome sequencing to determine how these aspects of evolution are entangled over time.

## CONFLICT OF INTEREST

None declared.

## Data Availability

The data that support the findings of this study are openly available in Data Dryad at http://doi.org/[tbd, reference number [TBD].
